# Dynamical transition of heat transport in a physical gel near the sol-gel transition

**DOI:** 10.1038/srep18667

**Published:** 2015-12-22

**Authors:** Kazuya U. Kobayashi, Noriko Oikawa, Rei Kurita

**Affiliations:** 1 Department of Physics, Tokyo Metropolitan University, Tokyo 192-0397, Japan

## Abstract

We experimentally study heat transport in a gelatin solution near a reversible sol-gel transition point where viscosity strongly depends on temperature. We visualize the temperature field and velocity field using thermochromic liquid crystals and polystyrene latex particles, respectively. During the initial stages of heating, we find that heat transport undergoes a dynamical transition from conductive to convective. Subsequently, during later stages, we observe that the transport dynamics are much more complex than conventional thermal convections. At the sample’s surface we observe the formation of stagnant domains, which lack fluid flow. Their formation is not due to the effects of local cooling. We determine that it is the dynamics of these stagnant domains that induce convective-conductive-convective transitions.

Gels are regarded as having highly functional properties: they are able to sustain their shape due to elastic polymer chain networks and also have the ability to transport materials. Typical examples are biomaterials such as protoplasm, agar, collagen (gelatin), the crystalline lens of the eye, etc. We focus here on physical gels whose components are held together by weak interactions such as hydrogen bonds^1^,^2^ such that they can be reversibly transformed into sol (liquid) states above a sol-gel transition temperature 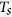. Despite being widely studied^3^,^4^,^5^,^6^,^7^, heat transport mechanisms in physical gels still remain unclear. One might think that conduction should be the dominant form of heat transport in a gel state due to high viscosity, while in a sol state flow plays a more important role. With very few experimental studies having been reported, the nature of thermal transport near the sol-gel transition temperature is non-trivial.

Heat transport in physical gels is also interesting from the viewpoint of non-equilibrium physics. For example, consider that heating the bottom of a fluid column produces a density gradient. Once the fluid’s thermal buoyancy overcomes its viscosity, Rayleigh-Bénard convection will occur. Rayleigh-Bénard convection has been studied extensively for its relationship with the formation of patterns, turbulent and chaotic flows, and in the Earth sciences^8^,^9^,^10^. For example, regular patterns of hexagonal convection cells, known as Bénard cells, will persist if the fluid’s viscosity is temperature dependent^11^,^12^,^13^,^14^,^15^. As a result, investigations of convections will typically use liquids such as silicon oil, honey, or golden syrup for their temperature-dependent viscosities^16^,^17^,^18^,^19^. Although many numerical simulations of complex phenomena, such as mantle convection, show the importance of the dependence between temperature and viscosity^20^,^21^,^22^, thus far there have been few studies of systems with a *strong* dependence. Here we propose physical gels as suitable materials for convective experiments since their viscosity is strongly temperature dependent, while their 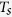 is conveniently near typical room temperatures. We investigate thermal transport in gelatin, a typical physical gel, around its sol-gel transition temperature by visualizing both temperature and velocity fields. Our interests lie in both its properties as a physical gel and more general non-equilibrium physics questions. In this Letter, we report a dynamical transition from thermal conduction to thermal convection in the initial stages of heating, and, in the latter stages, the observation of some extraordinary convective dynamics.

## Results

### Thermal transport at the initial stage

**Figure 1:**
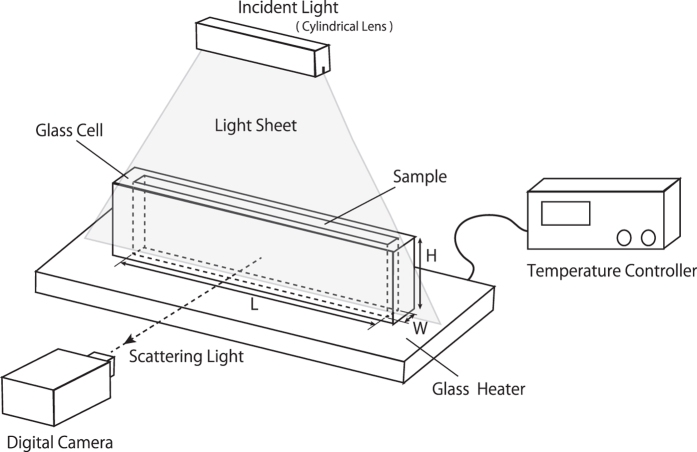
A schematic of our experimental setup. We use glass sample chambers of several different sizes. Their heights, lengths, and widths (H, L, W) are listed in our Methods section. We control the temperature at the bottom of the stage, while its top surface is free. We generate a light sheet using a cylindrical lens. Images are recorded by a digital camera.

First, we will briefly introduce our experimental setup, as described in Fig. 1. Our experimental setup consists of a glass sample chamber that is attached to a heating stage. We control the bottom of the sample’s temperature with the heating stage while the sample’s surface, at the top of the chamber, is free to equilibrate with room temperature. A standard air conditioner maintains a room temperature 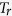 of 18–20 °C. We use a 5 wt% concentration gelatin solution, which has a sol-gel transition temperature 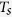 of 27.5 °C. Further experimental details can be found in the Methods section.

We begin by investigating the initial time evolution of the gelatin solution’s temperature field. In a chamber with dimensions (H, L, W) = (12 mm, 56 mm, 2.4 mm) and initially at a room temperature of ~20 °C, we load a solution of gelatin and micro encapsulated thermochromic liquid crystal (MTLC), at which point it is in a gel state. With the heating stage, we set the sample’s bottom temperature to *T*_*b*_ = 35 °C at time *t* = 0 s. Under these conditions, the sample’s top surface temperature eventually reaches and maintains *T*_*t*_ ~ 27 °C.

**Figure 2:**
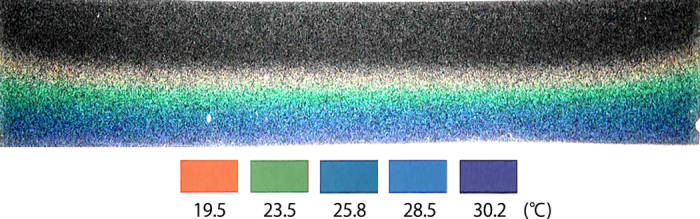
Temperature profile of the gelatin visualized by MTLC at *t* = 2 min. Temperatures are represented by different colors, as indicated by the color bar below the image. The temperature transports vertically and shows that conduction is dominant at the initial stage. The cell size is (H, L, W) = (12 mm, 56 mm, 2.4 mm). *T*_*b*_ = 35 °C and *T*_*r*_ = 20 °C.

**Figure 3:**
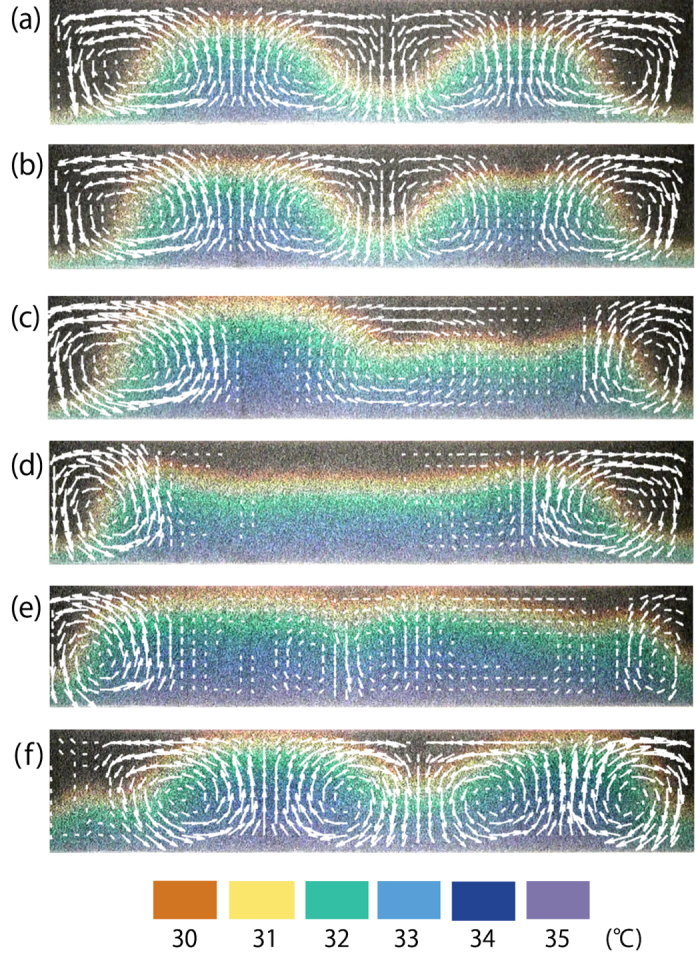
Time evolution of the velocity and temperature field visualized by MTLC in the later stages of Fig. 2. The color bar at the bottom corresponds to the range of temperatures. (**a**) t = 30 min, (**b**) t = 34 min, (**c**) t = 41 min, (**d**) t = 49 min, (**e**) t = 85 min and (**f**) t = 93 min. An undulation in temperature is formed and convection occurs (**a**). The shape of undulation is deformed with time (**b**) and the right undulation becomes flat (**c**). The flow virtually disappears and the temperature field becomes a trapezoidal (**d**). Due to thermal conduction, the isothermal lines align vertically toward the top surface (**e**). Then the convection is gradually formed again (**f**). The experimental conditions are same as that in Fig. 2. Under these conditions, 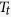 becomes 27 °C.

Figure 2 shows an image of the temperature field at *t* = 2 min. The colors correspond to the temperature, indicated by the color bar at the bottom of Fig. 2. Initially, the isothermal lines are almost flat, meaning that heat is transported by conduction. This seems natural, since the gel state’s viscosity is relatively high. However, the temperature profile cannot remain flat, and begins to undulate as shown in Fig. 3(a). We superimpose the velocity field in Fig. 3(a), clearly demonstrating the occurrence of thermal convection. Heating the gelatin, via conduction, reduces the gelatin’s viscosity, subsequently making the system unstable to thermal fluctuations. As a result, the dynamics of thermal transport changes from conduction to convection with time. These two phases of thermal transport, during the initial heating stages, differ from what is observed in water.

### Time evolution of thermal transport

Next, we investigate the time evolution of thermal convection in the later stages of heating (See Supplementary movie). We show the temporal change of the temperature field in Fig. 3(a–f) and superimpose the velocity field as a reference. The colors correspond to the temperature, as indicated by the color bar at the bottom of Fig. 3. The velocity field is obtained by corrected particle image velocimetry (PIV), which is described in detail in Methods. We note here that the error in our velocity measurements of MTLC is Δ*V* = 0.045 mm/s, which is too inaccurate for a detailed discussion of flow dynamics. Any background variation in intensity will severely bias our measurements, require a high pass filter (See Methods).

Figure 3(a) shows the convection after the dynamical transition from conduction. After a few minutes, in Fig. 3(b), one can see that the right side of the temperature undulation appears to split. The same side subsequently becomes flat, as shown in Fig. 3(c). Then, in Fig. 3(d), the whole profile becomes flat, excluding the boundaries. The velocity fields in Fig. 3(c,d) show that flow is greatly reduced before virtually disappearing. These results suggest a re-entrant dynamical transition from convection to conduction. As further evidence of thermal conduction, note that the isothermal aligns vertically toward the top surface, seen in Fig. 3(e). Interestingly, the instability reoccurs as convection gradually reforms, just as in the earlier stages (Fig. 3(f)). This series of changes can be described as a convection-conduction-convection transition.

**Figure 4:**
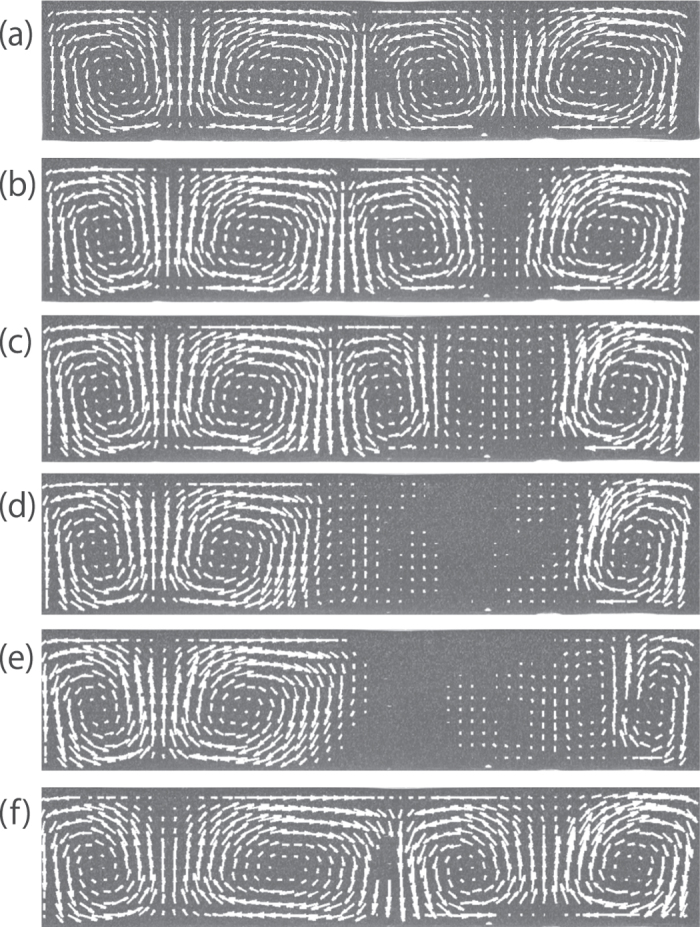
Velocity change over time in convective flows in gelatin visualized by PS latex. (**a**) t = 44 min. (**b**) t = 49 min. (**c**) t = 51 min (**d**) t = 54 min. (**e**) t = 57 min. (**f**) t = 62 min. First, a roll pattern appears (a). Then a stagnant domain is formed (**b**). The stagnant domain expands with time (**c**) and falls to the bottom by gravity (**d**). The macroscopic flow of the entire system drastically changes by sedimentation of the stagnant domain (**e**). After these events, the velocity profile gradually returns to the earlier stage (**f**). The cell size is (H, L, W) = (12 mm, 56 mm, 2.4 mm). *T*_*b*_ = 40 °C and *T*_*r*_ = 18 °C. Under these conditions, 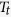 becomes 27 °C.

To better understand thermal transport at the later stages, we investigate the velocity fields with greater accuracy by using tracer particles. Instead of MTLC, we put polystyrene (PS) latex into the gel as tracer particles and the error in velocity measurements Δ*V* by PS latex becomes 0.015 mm/s, which is three times better than what can be achieved with MTLC. Figure 4(a–f) show the time evolution of the flow visualized by PS latex with the sample size (H, L, W) = (12 mm, 56 mm, 2.4 mm) at *T*_*b*_ = 40 °C, *T*_*t*_ = 27 °C and *T*_*r*_ = 18 °C. As shown in Fig. 4(a), a roll patten forms when convection occurs. The roll pattern varies over time in gelatin, which is distinct from that of convection in water. We find that a stagnant domain, without flow, is clearly visible at the top of the image, near the sample’s surface (Fig. 4(b)). When sedimentation occurs, due to gravity, it cancels out the upward convective flow, as shown in Fig. 4(c). As a result, the macroscopic flow at the centre has almost disappeared (*V* < 0.015 mm/s) in Fig. 4(d) and remains so for several minutes as shown in Fig. 4(e). Gradually, in Fig. 4(f), the velocity profile returns to the original roll pattern.

### Formation of stagnant domain

**Figure 5:**
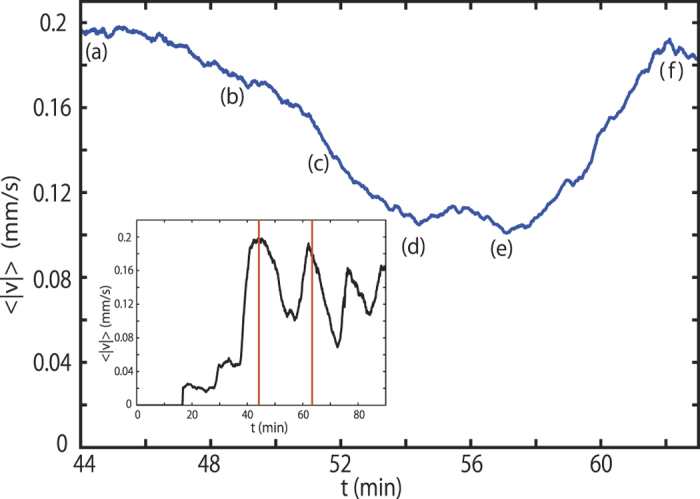
Average velocity near the top surface> 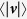 between *t* = 44 min and *t* = 63 min of the same experiment as Fig. 4. Inset figure shows the time evolution of 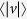 of the gelatin convection over the whole time.

Using our PIV measurements, we can quantitatively analyse the formation of the stagnant domain. We calculate velocities 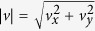 for every PIV box and average it with 3 layers of PIV boxes (about 3 mm) from the top surface. Figure 5 shows the time evolution of the average velocity near the top surface 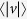 between *t* = 44 min and 63 min. Each point (a–f) in Fig. 5 corresponds to Fig. 4(a–f), respectively. 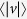 at the roll pattern is approximately 0.19 mm/s at (a) in Fig. 5. Then, the convection velocity decreases at (b) in Fig. 5, which corresponds to the formation and expansion of the stagnant domain. From macroscopic observation, sedimentation against the upward flow occurs at *t* = 51 min (Fig. 5(c)). At *t* = 54 min, 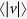 becomes half compared to the roll pattern, since the macroscopic flow is cancelled out by the sedimentation at (d) in Fig. 5. 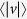 is then almost constant with time between *t* = 54 min and *t* = 57 min (Fig. 5(e)). After the flat region, 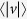 gradually increases to approximately 0.18 mm/s at (f) in Fig. 5 which is similar to the initial time (in Fig. 5(a)), when the roll pattern is first observed. We find that the temporal change of 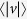 is consistent with macroscopic observations of the velocity field. Furthermore, we show 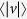 as a function of time over the whole measurement time as shown in the inset of Fig. 5. 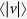 changes in an oscillatory fashion several times and these changes correspond to the series of stagnant region formation, sedimentation, cancellation of the macroscopic flow and the recovery of the roll pattern: these phenomena occur repeatedly.

## Discussion

Here, we combine results from both the temperature and velocity fields and consider the thermal transport mechanism in gelatin solutions near 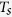. In the initial stage, the heat transport is governed by conduction, since the initial condition is a gel state. Then, the system becomes unstable due to thermal fluctuations and thermal convection occurs. In the latter stages, a stagnant domain is formed near the top surface, and the stagnant domain expands with time. Since the stagnant domain has little advection, it is cooled by the top surface and its density increases. As a result, sedimentation occurs cancelling out upward flow. Heat conduction becomes dominant again at this stage since macroscopic flow is small. Then the transition from conduction to convection occurs again due to instability, and convective effects gradually return to their behavior seen in the initial stage. We find that the convection-conduction-convection transition is repeated several times during our measurements. Here, it is worth noting that formation of the stagnant domain is reproducible, although the time and the position of the stagnant domain has not been determined, that is, the stagnant domain is sometimes formed in the left side of the chamber, sometimes formed in the right side and sometimes formed in both sides.

Now we can rule out another possibility for the velocity change. We may obtain a projection of 3 dimensional (3D) velocity in 2 dimensional (2D) PIV measurements, thus, the velocity could seem zero if the flow is perpendicular to the measurement surface. However, our observation is not due to 3D motion of the fluid. Width of light sheet is 6 mm, which is wider than sample thickness. We would observe both upward flow and downward flow along both sides of walls at the same time with 3D flow. In this case, the intensities would largely fluctuate. However, we do not observe any fluctuation of the intensities at the stagnant domain in a short interval when the sample size is (H, L, W) = (12 mm, 56 mm, 2.4 mm) (See supplementary movie). Thus we conclude that the stagnant domain is lack of flow rather than due to 3D flow.

Here, to understand the effects of boundaries on thermal convection, we investigate the convection of gelatin with sample chambers of several different sizes (See Methods). We find the formation of stagnant domain and the existence of sedimentation for all chambers and, most importantly, we observe this along the center of longer chambers. Our observations, therefore, suggest that the formation of the stagnant domain is not due to boundary effects. Although, there are experimental limitations such as thermal leak from the boundaries.

**Figure 6:**
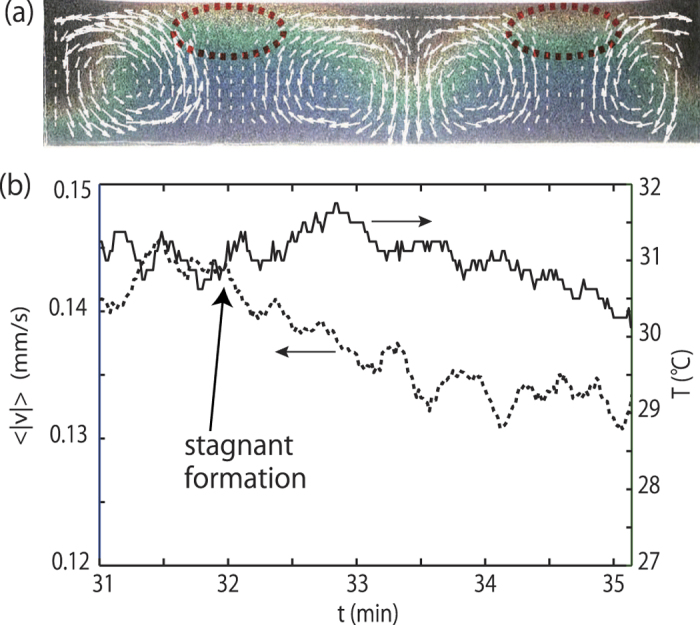
(**a**) Temperature and velocity fields visualized by MTLC at *t* = 33 min after the stagnant domain has formed. Temperature at the stagnant domain is the same as its surroundings. (**b**) Time evolution of 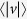 (dashed line) and *T* (solid line) during the formation of the stagnant domain. *T* remains constant when 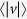 starts to decrease. The cell size is (H, L, W) = (12 mm, 56 mm, 2.4 mm). *T*_*b*_ = 35 °C and *T*_*r*_ = 20 °C. Under these conditions, 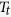 becomes 27 °C.

Finally, we discuss the origin of the formation of the stagnant domain, which should have higher viscosity. We investigate whether the formation of the stagnant domain occurs only close to 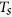. We set *T*_*b*_ = 50 °C, ensuring that the entire gelatin solution is in a sol state. As a result, we are unable to observe the formation of the stagnant domain. Then we cooled down the bottom temperature 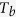 to 40 °C and find the stagnant domain is formed at the upward flow, which is similar to the experiment described in Fig. 4. Thus the stagnant domain formation occurs around 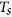 regardless of the experimental protocol. Alternatively, we only observe heat conduction when we set the bottom temperature below 30 °C, where the whole system is in a gel state. Thus we suggest that the formation of the stagnant domain is a specific feature of convection in physical gels only near the sol-gel transition temperature, where the viscosity dramatically changes. The viscosity of the physical gel is very sensitive to temperature around 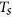: thus we measure the temperature of the stagnant domain just after forming the stagnant domain. Figure 6(a) shows temperature and velocity of flow in the gelatin at *t* = 33 min after the stagnant domain has formed. We find that the temperature at the stagnant domain is almost the same as its surroundings. Additionally, using the temperature and velocity fields visualized by MTLC, we show the time evolution 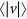 and *T* during the formation of the stagnant domain Fig. 6(b). We find that *T* remains constant, while 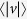 begins to decrease at *t* = 32 min. Our observations suggest that the formation of the stagnant domain, where flow disappears, is not caused by local cooling. We consider that those results suggest that it is necessary to consider other factors which affect viscosity. However, we do not have direct experimental results for the origin of the stagnant domain’s formation, thus necessitating further investigation.

To summarize, we experimentally study thermal transport in gelatin solutions by visualizing temperature fields and velocity fields near the sol-gel transition temperature. We observe a transition from conduction to convection at an early stage and observe complex dynamics. We find that these complex dynamics are induced by the formation of a stagnant domain and that the macroscopic flow changes due to sedimentation. Furthermore, we find that the stagnant domain’s temperature is not lower than its surroundings when it has just formed. From these results, it seems that the formation of the stagnant domain is not solely caused by temperature-dependence on viscosity. Further investigations are required to better understand the formation of the stagnant domain.

## Methods

### Sample

We use gelatin purchased from Wako Pure Chemical Industries Co., Ltd. and make a 5 wt% concentration gelatin solution using pure water as a solvent. The sol-gel transition temperature of gelatin is approximately *T*_*s*_ = 27.5 °C for 5 wt%, as determined by the falling ball method and it is consistent with ref. 7. We measure the viscosity of gelatin by steady shear flow measurements with shear rate *γ* = 0.1 s^−1^, which is similar to that under the convection flow of gelatin. We find 5 Pa·s at 27 °C and 0.05 Pa·s at 40 °C. During the experiment, viscosity at the top of the sample is roughly 100 times greater than at the bottom. The evaporation of water during our experiment is negligible (<0.1%).

Our experimental set up is roughly sketched in Fig. 1. We make sample chambers using glass and we prepare several sizes of the sample chambers, where height, length and width (H, L, W) are (12 mm, 56 mm, 2.4 mm), (12 mm, 136 mm, 2.4 mm), (12 mm, 56 mm, 3.6 mm), (12 mm, 56 mm, 4.8 mm) and (12 mm, 56 mm, 7.2 mm). Thermal conductivity of glass is about 0.6 W/mK at the room temperature, which is comparable to that of water (~0.6 W/mK). We control the temperature of the bottom surface using a temperature controller (S100, Blast Co.) and keep the top surface free. During our experiments, the room temperature 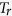 is set to 18–20 °C with a standard air conditioner.

We set the bottom temperature 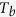 to 35 °C or 40 °C in order for the top surface temperature 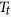 to become ~27 °C, which is just below 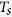, as measured independently using a thermometer. We use a cylindrical lens for generating a light sheet for incident light. The width of the light sheet is about 6 mm. We record the images with a digital camera (Model HC-V520M, Panasonic Co.).

### Visualization of temperature and velocity fields

We obtain images with 1834 pixels × 386 pixels, which corresponds to 56 mm × 12 mm. We divided an image by 32 pixels × 32 pixels and use Fast Fourier Transform (FFT) based cross correlation algorithms for PIV. A time separation of two images for PIV is fixed to 1 s, which is optimized for the maximum speed of the thermal convection.

We use micro encapsulated thermochromic liquid crystal (MTLC) purchased from the Japanese Capsule Products Company (density: 1.01 g/*cm*^3^) for visualization of the temperature field^23^,^24^. We determine the temperature from the color and the error in temperature Δ*T* = 0.1 K. To determine the flow from MTLC images, we need to remove the temperature profile using a high pass filter before using the usual PIV method^25^. Due to this correction, a peak of a correlation becomes broader and it leads to larger errors in velocity, of Δ*V* = 0.045 mm/s. Thus, MTLC images can only provide an approximate velocity. For more accurate velocity measurements, we put polystyrene (PS) latex (density is 1.05 g/*cm*^3^) into the sample as tracer particles, instead of MTLC, for quantitative analysis of the flow. We determine that Δ*V* of PS latex measurements is 0.015 mm/s, three times better than using MTLC images.

## Additional Information

**How to cite this article**: Kobayashi, K. U. *et al.* Dynamical transition of heat transport in a physical gel near the sol-gel transition. *Sci. Rep.* **5**, 18667; doi: 10.1038/srep18667 (2015).

## Supplementary Material

Supplementary Movie

Supplementary Information
