# Supplementary material for: Dynamical transition of heat transport in a physical gel near the sol-gel transition
Source: Sci Rep. 2015 Dec 22;5:18667. doi: 10.1038/srep18667 (PMC4687040; doi:10.1038/srep18667)
Supplement: Supplementary Information [file srep18667-s2.doc]

Supplementary

TITLE: Dynamical transition of heat transport in a physical gel near the sol-gel transition.

Authors: Kazuya U. Kobayashi, Noriko Oikawa, Rei Kurita

(Smovie) Velocity change over time in convective flows in gelatin visualized by polystyrene latex particles between t = 44 min and t = 62 min. The speed of this movie is 30 times faster than real time. This experiment is same as Fig. 4 in the main manuscript. The cell size is (H, L, W) = (12 mm, 56 mm, 2.4 mm).
